# Sociocultural Dimensions of Children’s Physical Activity in Contemporary Pastoralist Maasai Society

**DOI:** 10.3390/ijerph18168337

**Published:** 2021-08-06

**Authors:** Xiaojie Tian, Tetsuhiro Kidokoro, Francis Mundia Mwangi

**Affiliations:** 1 Faculty of Health and Sport Sciences, University of Tsukuba, Tsukuba 305-8574, Japan; 2 Faculty of Sport Science, Nippon Sport Science University, Tokyo 158-8508, Japan; kidokoro@nittai.ac.jp; 3 Department of Physical Education, Exercise & Sports Science, Kenyatta University, Nairobi 43844-0010, Kenya; mwangi.francis@ku.ac.ke

**Keywords:** pastoralist Maasai children, physical activity, sociocultural roles, subsistence work, outside school activities, gender differences

## Abstract

Children’s physical activity (CPA) in low- and middle-income regions has received increasing attention, but research is still very limited. This study explores the CPA in contemporary pastoralist Maasai society in rural Kenya by considering its sociocultural dimensions. The physical activity of 25 children (15 girls and 10 boys) was documented with mixed methods, including an epidemiological assessment of the CPA and semi-structured interviews with the targeted children regarding their daily activities. These methods were integrated with the ethnographic data on children’s socialization in the same area. Results showed a very high level of moderate-to-vigorous physical activity (MVPA) of these children with significant gender differences especially outside school. Children reported their continued social participation in local gender–age labor divisions outside of school. As their activities outside school strongly contributed to their empirical learning of local knowledge and skills, a high MVPA plays an active role in enhancing the children’s ability to access and manage livestock and different natural resources. Findings from this study first show that the CPA is not merely physical, but also has significant sociocultural meanings in the process of in situ learning of local wisdom. We call more attention to children’s social roles in future investigations of CPA among less examined populations.

## 1. Introduction

### 1.1. The Under-Investigated Children in Current Physical Activity Discussions

Since the call for greater attention to the pandemic of global physical inactivity [1,2,3,4,5,6], an increasing number of studies have examined children’s physical activities (CPA) in different regions across the globe over the last two decades. These studies identified strong correlations between CPA and physical, emotional, and intellectual development in early human life and its continued influence on wellbeing over the course of a human life, e.g., [7,8]. Along with this trend, new investigations of CPA across various regions have emphasized its diverse forms in different sociocultural and ecological contexts [9,10,11,12,13]. This study focuses on children in the pastoralist Maasai society in rural Kenya, aiming to understand the conditions of CPA in contemporary Maasai society by considering its sociocultural aspects.

To better understand CPA and its diversity, two main limitations have been well addressed in recent literature: first, the unbalanced publication coverage of study locations; second, the narrow understanding of CPA conditions outside of school, especially among less studied populations. For the former, recent systematic reviews focusing on physical activities have detected regional and national gaps in the existing studies [3,10,13,14,15]. For instance, by focusing on the current trends of physical activity studies in relation to regional and national economic and developmental conditions, Varela et al. [13] found that the most covered populations are in Europe, and the least investigated populations are in Southeast Asian and African regions. In addition, the well-investigated populations are concentrated in high-income countries, whereas low- and middle-income countries (LMICs), including many Asian and African countries, have yet to receive sufficient attention. Lambert et al. [15] emphasized that such location bias should not only be perceived as a research gap; it involves complex social, political, and public health contexts, and hence, the results should not be generalized under one standard.

To extend this discussion on study location bias, the second challenge in the existing literature is that very limited attention has been paid to the diverse sociocultural contexts in LMICs. Current studies that focus on people’s physical activity in LMICs are still limited in number, with an inadequate understanding of local sociocultural determinants and few evidence-based details concerning physical activity [1,3,10]. As Strain et al. [16] emphasized, physical activity as a complex human behavior exists in various domains of life, such as the forms of working and playing in different social environments. Their country-level analysis of domain-based activities demonstrates that work-related activities contribute more to the high physical activity (PA) level of adults in LMICs.

It should be noted that many LMICs are characterized by diverse lifestyles and sociocultural contexts, particularly compared to high-income countries. When focusing on children, the CPA conditions in less focused areas do not rely only on school-based activities. Especially in rural areas, populations tend to have limited school education resources but are rich in sociocultural practices. As many scholars have illustrated, school-based physical education in the sub-Saharan African region is not yet well managed, with very limited infrastructure and human resources [17]. In such regions, the children’s outside school activities determine much of their daily energy expenditure and physical development [18,19]. For example, following the discoveries of forager Hadza children’s self-provision of daily caloric needs through daily foraging activities with peers [20], Froehle et al. [18] further investigated the characteristics of Hadza children’s daily physical activity and its relation to the net caloric returns from their activities. Their findings identified clear gender–age differences in work-related activity participation and caloric expenditure and gain among these children during their work. Their study draws more attention to the relationship between children’s social participation and physical activity in small-scale societies.

### 1.2. Ascertaining Maasai Children’s CPA from a Biosocial Approach

To date, different theoretical concepts have been developed for a holistic understanding of the physical activity and daily life of different populations. For instance, the ecological model takes physical activity at the individual level as a consequence of different styles of living that are continuously influenced by both physical and social environments [21]. Physical environmental factors, such as the designed and built living space and the distance to the natural environment, may induce physical constraints that limit the accessibility of the targeted population’s accessibility to these areas, thus potentially confining their daily physical activity levels. Social environment factors, such as social norms, social support, and cultural practices in different living contexts, including home, school, and the workplace, may also impact individuals’ daily physical activity levels. These determinants are crucial and vary from one society to another; thus, careful analysis is required [1]. The ecological model has been evaluated highly for its potential to enrich our current understanding of the epidemiological findings, by questioning the causes and processes in complex living forms [1,21].

Applying the ecological model to the investigation of CPA in LMICs enables a holistic understanding of CPA by considering its sociocultural dimensions. This can be achieved through a combination of both quantitative and qualitative approaches to CPA. In addition to the quantitative monitoring of CPA levels, qualitative documentation of the contents of activities, mobilities, and companions of individual children at the daily activity level as well as crucial sociocultural factors, such as the local living environment and the social roles of children, could be effective in capturing the concurrent sociocultural dimensions of CPA in a certain society. Thus, this study combines both quantitative and qualitative methods to understand CPA in Maasai society. In addition to the epidemiological physical activity levels of individual children, the ethnographic documentation of the contents of children’s daily activities, local living environment, and social support are also considered crucial factors for a holistic examination of the sociocultural dimensions of CPA in modern Maasai society.

### 1.3. Pastoralist Maasai and Their Children

The Maasai are pastoralists who inhabit a vast area of arid and semi-arid savanna land in southern Kenya and northern Tanzania. Since the 1960s, the Maasai have experienced lifestyle changes under several external pressures, including recurrent droughts, government-initiated land subdivisions and privatization, wildlife-centered tourism, and agriculture-centered economic development, all of which devalue the local practice of pastoralism [22,23]. In Kenya, the Maasai’s access to pasturelands has been restricted, making them increasingly dependent on market-driven goods and public services [24]. Notwithstanding these changes, livestock keeping has persisted as the primary sustenance resource with sociocultural significance supporting local life.

Similar to many other pastoralist societies in East Africa, gender and age labor divisions play important roles in assisting the continuation of pastoralist ways of living. In Maasai society, men and women have different social roles. Men focus on livestock and pastureland management, while women perform household chores, including house construction, food preparation and division, and childcare. These tasks are further divided according to the age and life stages of individuals.

A man’s life consists of three stages: boyhood, young manhood (*ilmurran*), and elderhood [25]. Boys begin tending to livestock from an early age, through different tasks such as milking and taking care of juvenile livestock at home. Around the age of 10, when they can identify their father’s livestock and are able to control the movements of the herds, they are entrusted with greater responsibility and positively supported by local communities with day trip herding tasks [26]. Boyhood lasts until around the age of 15. After receiving circumcision, boys become young men, forming their own age group, and are entrusted with the responsibility of securing the safety of the people and livestock in the village. The *ilmurran* period lasts for approximately 15 to 20 years and involves tasks that require high energy expenditures, such as long-distance herding during the dry season and the construction and maintenance of seasonal water points. The physical strength built from boyhood plays an important role during the early adult stage. Finally, when young men enter their elderhood, they become more involved in political issues such as livestock trading and land ownership decisions.

A woman’s life stages mainly consist of a girlhood stage and a married womanhood stage. From an early age, girls begin to participate in household chores such as milking livestock and cleaning the house. As they grow up, they are gradually entrusted with more energy-intensive tasks such as collecting firewood and fetching water. Unlike men, women do not form their own age group. With less political power in local society, girls and adult women usually form collaborative relationships when performing different daily tasks [27]. Nowadays, as formal education has become compulsory and is highly valued in Maasai society, adults are adjusting their daily roles to support children’s school participation. Meanwhile, social participation through livestock tending and household chores is continuously practiced by children and is also highly valued by the local society as children gain local wisdom through these activities.

Since Kenya’s independence in 1963, formal education has been brought to pastoralist societies by both the government and missionaries through a top-down approach that neglects careful consideration of local socioeconomic conditions and lifestyle [28]. While pastoralists have gradually come to view formal education as an important alternative for them to cope with external social and environmental uncertainties, several challenges still exist, including poor financial and infrastructural conditions, deficient human resources, limited understanding of local lifestyles, constrained opportunities for children, and limited access to high-quality education [29,30]. In this context, pastoralist ways of living with their own educational philosophies still play an active role in providing wisdom for young generations to manage their wellbeing in this unpredictable environment [31].

The highly specialized knowledge that pastoralists have developed includes, but is not limited to, their ways of naming and classifying the livestock and the biota and the skills with which they manage them according to the seasonal changes in the rangeland and the savanna ecosystem [32,33,34]. For children in these societies, competence also means being skillful in managing the everyday conditions of livestock and the natural surroundings. This process requires intimate and sensory interactions between children, livestock, and the biota from an early age. Notably, in contrast to classroom learning, this form of in situ learning and socialization of children entails considerable energy expenditure and rich body movements at different life stages.

## 2. Study Area and Research Methods

This study was conducted in a Maasai village located on the eastern edge of Kajiado County in southern Kenya. This area has an arid and semi-arid savanna climate and is well known for its rich wildlife, for instance, lions, elephants, and giraffes. Local people have increasingly adapted to diverse forms of economic activities, such as tour guides and small businesses, while livestock grazing as the main subsistence activity is continuously practiced. In the study area, the Maasai living environment generally includes three spaces: home, village, and forest. The home covers an area of approximately 5000 m^2^ and consists of a well-fenced compound and a reserved grazing area surrounding the compound. People belonging to extended families usually live together in the same compound as their livestock. Collectively, the residential area of the village is approximately 12 km^2^, consisting of all the homes, a shopping town, and a primary school. The local primary school consists of a main campus approximately 4 km^2^ in size, next to which is an extended playground covering an area of approximately 2 km^2^. The forest refers to an area of approximately 200 km^2^ surrounding the village that covers hills and rangelands for seasonal grazing and riparian and swamp areas for farming. These areas are all living environments in which children visit with their peers during different daily activities.

This study combines the epidemiological approach with an ethnographic approach to understand the sociocultural dimensions of Maasai children’s physical activities in their daily lives. More than 85% of primary-school-age children in this area attend school with positive support from their families. The physical activity of individual children was measured using an epidemiological approach in February 2020, which was during the dry season with an approximate temperature of 16 °C to 30 °C. Through the collaborative research efforts shared by all authors (details are in “Author Contribution” section), the physical activity and sedentary behaviors of 25 school children (10 boys and 15 girls) aged 9–12 years in the same village were measured using three-axis accelerometers (ActiGraph wGT3X-BT, LLC, Pensacola, FL, USA). This method has been shown to be valid for measuring PA and sedentary behavior (SB) in children [35,36]. We randomly selected children who were in good health conditions and regularly attended the primary school in the local area. Before the data collection, informed consent was obtained from both the guardians and the participating children with a detailed explanation of this research project. All children and their guardians (including both parents and teachers) agreed to undergo the accelerometer assessments for a period of seven days (Monday to Sunday). This period covered five school days, from Monday to Friday, and two holidays: Saturday and Sunday.

Physical activity data were collected in 15 s epochs. Non-wear time was defined as a period of ≥60 min of continuous zero counts, as recorded by the ActiGraph [37]. Only participants with ≥10 h of wear time per day for a minimum of four days (including at least one weekend day) were included in analysis [38]. Evenson’s cut-off points [35] were used to categorize the activities into three levels: SB, <101 counts per min (CPM); light-intensity PA (LPA), 101–2295 CPM; MVPA, ≥2296 CPM, which consists of moderate-intensity PA (MPA), 2296–4011 CPM; vigorous-intensity PA (VPA), ≥4012 CPM. The collected data were analyzed using ActiLife software (version 6.13.3; ActiGraph, LLC, Pensacola, FL, USA). For the data analysis, independent *t*-tests were performed to compare differences in PA (i.e., MVPA level) and SB between boys and girls. A chi-square test was used to examine the differences in daily activities between boys and girls. Statistical analyses were performed using SPSS version 24 (SPSS, Inc., IBM, Armonk, NY, USA). During the analysis, weekday and weekend data were separated according to the daily schedule of the school and daily routine in the study area. Weekday data were divided into three sets: before school (6:00 am–7:59 am), during school (8:00 am–4:30 pm), and after school (4:31 pm–9:00 pm) according to the local school schedule. The weekend data were simply divided into two sets: morning hours (6:00 am–11:59 am) and afternoon–evening hours (12:00 pm–9:59 pm) based on the local weekend routine.

After the accelerometer data were collected and confirmed as validated, children were requested to provide feedback on the details of their daily activities during the period for which they carried the machine through semi-structured interviews. They were conducted through visual meetings with each participant with the help of a local research assistant. These questions included the content of activities, the time (i.e., weekday, weekend, or both; morning, afternoon, or throughout the day), location (i.e., school, home, or other locations), companions present during each mentioned activity, and the continuation of these activities before and after the accelerometer assessment. For example, we first asked, “What did you do during the morning hours of school days while carrying the accelerometer?” After the children listed all the activities as feedback to this question, we inquired further about each activity by asking where and with whom he/she performed that activity. We then confirmed whether these activities were practiced before this data collection as a daily routine.

The ethnographic research of the Maasai children’s daily routine and social participation in the same area was continuously conducted by the first author for an intermittent period of 15 months of participant observation from 2013 to 2018. During the long-term fieldwork, a total of 218 h of dawn-to-dusk focal child observation was conducted on 13 children (nine boys and four girls, aged 2–12 years) with attention given to the details of individual children’s daily activities, their mobilities, and the daily interactions of each child with other social members. In addition, ethnographic data from long-term observations of labor divisions and social norms for child rearing were used to explain the sociocultural roles of children in daily activities. These data, together with the epidemiological findings, are used to investigate the diverse sociocultural meanings of physical activities for children in contemporary Maasai society.

## 3. Results

### 3.1. Physical Activity of Maasai Children

**Figure 1:**
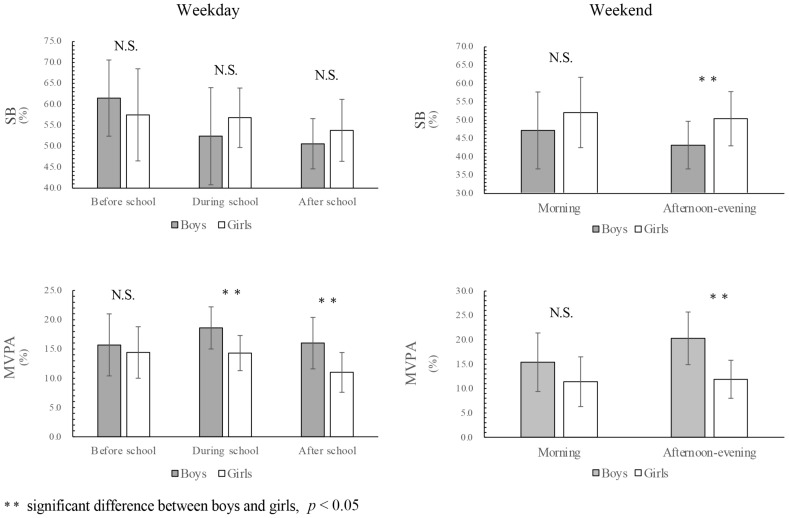
SB and MVPA with gender differences on weekdays and weekends.

**Figure 2:**
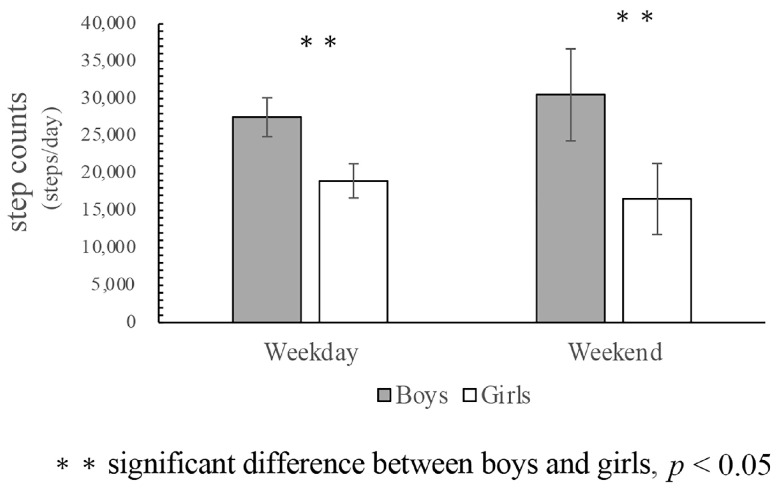
Step counts with gender differences.

Figure 1 shows the results of physical activity of the 25 Maasai children, separated by gender, during the one-week period. During the weekday, the MVPA levels of both boys and girls were high, and both met the 60 min of physical activity guidelines of the World Health Organization (WHO). However, there were significant gender differences in children’s MVPA levels during and after school. During the weekend, boys’ MVPA levels increased further, and their level of SB decreased sharply. Significant gender differences were also found in both sedentary behavior and MVPA levels during the afternoon-evening time segment. Together with the number of step counts (Figure 2), these data emphasize the high MVPA level of Maasai children.

### 3.2. Daily Activities in and Outside School

On school days, all children go to school on foot. As children are requested to have lunch at home, they need to travel between home and school a total of four times daily, which contributes considerably to the high MVPA during school days. During the period of data collection, the local primary school started at 8:20 a.m. and finished at 4:30 p.m. The teachers also set morning hours from 7:00 a.m. until the start of the remedial period. Following this schedule, children usually arrived at school before 7:00 a.m. They had one physical education class per week and had free play and game time from 3:10 p.m. to the end of the school on Mondays, Tuesdays, and Thursdays. On Friday afternoons, all children participated in school cleaning and toilet washing. According to them, during this task, girls and boys had different responsibilities: girls wiped the floors, desks, and chairs, and boys fetched water and assisted girls with washing and cleaning. Inside the school, the open playground, together with the open spaces in between two classroom buildings, form the main sports facilities and are used for PE class and playing activities. Children mentioned that there were footballs, handballs, and skipping ropes in the school, but they were limited in number. Both boys and girls usually made their own sandbags with broken socks or fabric wastes, sometimes filling them with sand or dried grass.

**Figure 3:**
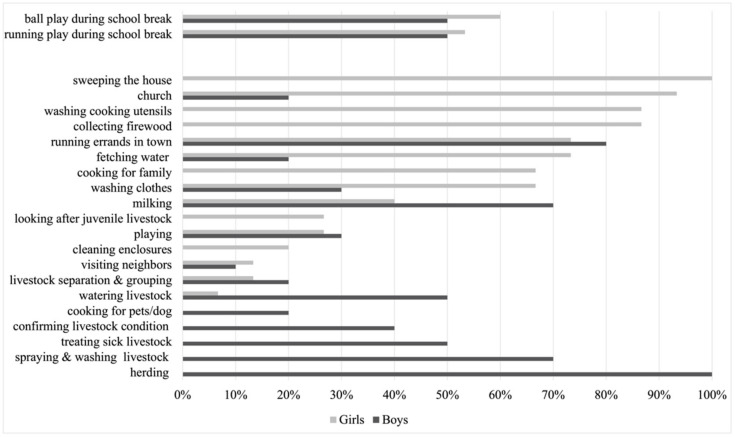
Daily activities of Maasai children, distinguished by gender.

Figure 3 summarizes the activities mentioned by the children with gender differences. Because children share the same school schedule with similar learning tasks on school days, only school play activities were listed. There are two designated time periods during which school children can play: play time and game time in the afternoon, as mentioned above, as well as brief recesses between classes, including lunch breaks. Both girls and boys mentioned ball games and running as play activities. The ball games included football, netball, and sandbag-throwing games. The limited types of children’s play activities inside school may be due to the limited school recreational facilities. It may also relate to the ways in which children self-report their play activities inside the school. As local traditional play is commonly practiced by children at home but is less encouraged in school, there is a possibility that children avoid mentioning them. The four trips between home and school, ball playing, running, and the cleaning tasks may collectively contribute to the high level of MVPA for both girls and boys during school days. Moreover, the activities of girls and boys were quite similar. Thus, the gender differences in physical activities among these children are more likely to be more related to the activities that children are involved in outside of school.

Children mentioned that their activities outside school included different types of subsistence work and chores, social gatherings, and play. Subsistence chores included household and livestock tasks. The household tasks included fetching water, cooking for family, cooking for dogs (pets), washing clothes and utensils, cleaning the house, firewood collection, and running errands such as shopping in town or sending messages for adults to neighbors or relatives. Among these activities, girls reported that their participation mainly involved household tasks. Unlike girls, the main tasks mentioned by the boys were related to livestock management. In addition to the aforementioned subsistence tasks, about one-third of the children also mentioned their play activities after school. These included hide and seek, running and chasing, ball games, and tree climbing.

### 3.3. Frequency, Locations, and Companions

The frequency, location, and companions that children described in their activities outside of school are listed in Table 1. This information provides a better understanding of the mobility of these children and how they conduct different activities in daily life. First, the mentioned activities were continuously practiced before and after the accelerometer assessment period as routines with different frequencies: daily, weekdays only, weekends only, or occasionally. In some instances, this means that the reported activities were only conducted when necessary, such as when instructed to by adults. Regarding location, children performed subsistence chores and household work, traversing all the living environments, including schools, homes, villages, and forests. Children were conditionally entrusted by local adults for their ability, depending on their age, to move either independently or with peers across different living areas.

With the information on the contents, frequency, locations, and companions of different types of activities of the Maasai children (see Table 1), we could further identify what type of activity contributed to their high MVPA and explain gender differences in time and space. On weekdays, active play in school, subsistence work and chores outside school, and walking between school and home inside the village were important determinants of high MVPA.

At weekends, active participation in chores and walking around the village and in forest areas contributed to children’s high MVPA. The gender differences in MVPA throughout the week are strongly related to children’s social participation in local labor divisions, such as boys’ livestock tending and girls’ household chore participation. Such gender differences also reflect the characteristics of children’s companions during different subsistence tasks. To further explore the sociocultural meanings of CPA in Maasai society, in Section 4, we integrate the observations from the ethnographic documentation of the skills and knowledge that children developed through two representative tasks, herding livestock for boys and collecting firewood for girls.

## 4. Discussion

As noted in the introduction, the physical activity level of people from non-Western and non-industrialized societies has received less attention in current research, with very limited investigation of the sociocultural dimensions of the CPA in these regions [3,9,10]. Focusing on CPA and its sociocultural aspects among these populations helps contribute to a better understanding of the diverse impacts of physical activity in the growth of children across the globe. The results from this study show a significantly high level of MVPA among pastoralist Maasai children, aged 9 to 12 years, attending local primary school with clear gender differences. From the content of the children’s self-reported daily activities, their participation in organized play and sports activities is limited in variety, and such activities only occur during school recesses. Outside of school, the children continuously participate in livestock-related chores and household work. These activities are practiced with gender differences and are accompanied by active movement on foot across different living spaces. Therefore, besides organized play or sports in the school setting, outside school activities, especially children’s participation in different subsistence tasks, contribute to their high level of MVPA. These findings call attention to the correlation between active CPA and children’s outside school activities in small-scale societies in MLICs.

Second, the authors intend to address the physical activity of Maasai children with their in situ local knowledge and skill learning from the perspective of an ecological model [1]. As has been discussed by many scholars in the fields of anthropology and developmental psychology, the work or chore participation of children in many small-scale societies as part of their family life has significant developmental functions, as children learn local skills and develop social relations [39,40]. To achieve competency in pastoralist societies, children need to generate local skills and knowledge by empirically interacting with livestock and biota through different activities from an early age [26,31,32]. Herding and firewood collection are the two primary chores that school-age children continuously performed during the non-school days in the study area. These activities required the children to travel long distances and directly interact with livestock and biota in different environments. During herding, the boys would spend a full day across a distance of 5–20 km with their livestock according to the seasonal conditions of the grasslands and water resources. For this task, they needed to frequently confirm the location and health conditions of individual livestock, track the movements and directions of the herds, avoid mixing them with other herding groups, and control and guide the speed and direction of the herds to the right pasture lands at the right time. After a daytrip of herding, they also needed to ensure that all the livestock returned home safely with no injuries and full stomachs. In addition, the success of the daytrip, herding also required the application of rich local knowledge of plants and wild animals, for instance, to identify different edible plant species seasonally for different types of livestock and to read the tracks of wild animals to avoid encounters with dangerous animals while herding. Similarly, girls moved approximately 3–6 km away from the village for firewood collection [27]. Through this task, they empirically gained local knowledge of different types of tree species, such as local names, locations, and seasonal availability. In this study, as all the boys mentioned their participation in herding and girls in firewood collection as regular routines, for them, the high levels of MVPA play a sociocultural role of continued in situ learning of local skills and knowledge of the livestock and natural surroundings.

Above all, the high level of CPA for Maasai children is not only related to their individual health and development. It also has sociocultural significance linking them to the sensory local knowledge learning and skill acquisition. From this perspective, the CPA of children of different ages should be understood within the developmental niches of the children, with both biological and sociocultural significance. This perspective is not limited to Maasai children, but should be taken into consideration when investigating the CPA in other small-scale societies.

Finally, two limitations are noted in this study. First, as the answers from the children were quite brief regarding their school physical activities, it was difficult to further explain the details of local physical education conditions. Second, as we did not collect caloric data, it is difficult to assess the energy balance of the targeted children. As the current study focused more on the relation of CPA and children’s daily activities, our findings are not influence by these two factors. However, as local people are experiencing social changes, changes in these two factors may consequently influence CPA and children’s social participation in the future. It would be interesting to include them in further investigations to gain a more comprehensive understanding of the biosocial development of these children in a changing world.

## 5. Conclusions

The findings of this study first emphasize that the children in small-scale societies are usually physically active in their daily lives. Although they are gradually experiencing lifestyle changes, such as more time spent in school, their high levels of MVPA can be sustained when they continuously perform their social roles outside of school. Second, together with the previous findings regarding children in hunter–gatherer societies [18], this study emphasizes that the high levels of MVPA exhibited in these children is strongly linked to their daily routines and social participation outside the school context. Moreover, it is worth noting that children in small-scale societies learn local knowledge and gain cultural identities by performing their social roles in daily activities. This suggests that children’s physical activity plays a crucial role in the process of daily in situ learning, which requires further detailed investigation.

The current WHO guidelines regarding suggestions for the improvement of children’s inactivity, for instance, are usually based on the findings in Western and industrialized nations. Under this situation, the diverse conditions of children in small-scale societies have largely been overlooked. This omission may induce inadequate physical educational program design at both the national and international levels. Adapting and implementing national or international education policies in these less investigated societies without knowing the CPA conditions outside of school may have an unexpected negative influence on these children—not only in terms of their health but also their ability to gain sociocultural competence in local skills and knowledge. In conclusion, it is recommended that more attention be paid to the sociocultural dimensions of CPA in less investigated societies, through detailed ethnographic observations of children’s daily lives both in and outside of school.

## Figures and Tables

**Table 1:** Time, location, and companions for different activities.

Activities	Routine	Location	Companion (s)		
			None	With Children	With Adults
playing ball (in school)	weekdays	school	-	children	-
running	weekdays	school	-	children	-
herding	weekends	forest	-	boys	-
collecting firewood	weekends	forest	-	girls	female adults
watering livestock	daily	village	-	brothers/sisters	-
fetching water	daily	village	-	brothers/sisters	mother
spray and wash livestock	occasionally	village	-	brothers	father/mother
visiting neighbors	weekends	village	alone	-	-
running errands in town	daily	village	alone	sisters	-
confirming livestock condition	daily	home	alone	brothers	-
looking after juvenile livestock	daily	home	-	brothers	-
treating sick livestock	occasionally	home	alone	-	father
milking	daily	home	-	brothers/sisters	mother
cooking for pets/dog	daily	home	alone	-	-
livestock separation and grouping	daily	home	alone	brothers/sisters	mother
washing clothes	daily	home	alone	sisters	-
washing cooking utensils	daily	home	alone	sisters	-
sweeping the house	daily	home	alone	sisters	-
cleaning enclosures	weekends	home	-	brothers/sisters	mother
cooking for family	daily	home	alone	sisters	mother
playing	daily	home	-	children	-
church	weekends	village	-	children	all adults
